# Challenges in multidisciplinary cancer care among general surgeons in Canada

**DOI:** 10.1186/1472-6947-8-59

**Published:** 2008-12-22

**Authors:** Anna R Gagliardi, Frances C Wright, Dave Davis, Robin S McLeod, David R Urbach

**Affiliations:** 1 Sunnybrook Health Sciences Centre, 2075 Bayview Avenue, Toronto, Ontario M4N3M5, Canada; 2 Association of American Medical Colleges, 2450 N Street NW, Washington, DC 20037-1127, United States; 3 Mount Sinai Hospital, 600 University Avenue, Toronto, Ontario M5G1X5, Canada; 4 University Health Network, 200 Elizabeth Street, Toronto, Ontario M5G2C4, Canada

## Abstract

**Background:**

While many factors can influence the way that cancer care is delivered, including the way that evidence is packaged and disseminated, little research has evaluated how health care professionals who manage cancer patients seek and use this information to identify whether and how this could be supported. Through interviews we identified that general surgeons experience challenges in coordinating care for complex cancer patients whose management is not easily addressed by guidelines, and conducted a population-based survey of general surgeon information needs and information seeking practices to extend these findings.

**Methods:**

General surgeons with privileges at acute care hospitals in Ontario, Canada were mailed a questionnaire to solicit information needs (task, importance), information seeking (source, frequency of and reasons for use), key challenges and suggested solutions. Non-responders received up to three reminder packages. Significant differences among sub-groups (age, setting) were examined statistically (Kruskal Wallis, Mann Whitney, Chi Square). Standard qualitative methods were used to thematically analyze open-ended responses.

**Results:**

The response rate was 44.2% (170/385) representing all 14 health regions. System resource constraints (60.4%), comorbidities (56.4%) and physiologic factors (51.8%) were top-ranked issues creating information needs. Local surgical colleagues (84.6%), other local colleagues (82.2%) and the Internet (81.1%) were top-ranked sources of information, primarily due to familiarity and speed of access. No resources were considered to be highly applicable to patient care. Challenges were related to limitations in diagnostics and staging, operative resources, and systems to support multidisciplinary care, together accounting for 76.0% of all reported issues. Findings did not differ significantly by surgeon age or setting of care.

**Conclusion:**

General surgeons appear to use a wide range of information resources but they may not address the complex needs of many cancer patients. Decision-making is challenged by informational and logistical issues related to the coordination of multidisciplinary care. This suggests that limitations in system capacity may, in part, contribute to variable guideline compliance. Further research is required to evaluate the appropriateness of information seeking, and both concurrent and consecutive mechanisms by which to achieve multidisciplinary care.

## Background

Cancer is a leading cause of premature death in many countries, but medical knowledge is thought to be sufficiently advanced such that one third of cancers could be prevented, a further one-third cured given early diagnosis, and the remainder effectively treated if management consistently complied with existing evidence-based standards [1]. Population-based studies from Canada, Australia and the United States have demonstrated that practice often differs from guidelines for cancer [2-8]. While many organizational and system-level factors can influence the way that cancer care is delivered, including the way that evidence is packaged and disseminated, little research has evaluated how health care professionals who manage cancer patients seek and use this information to identify whether and how this could be supported [9-16].

Research on information seeking among general practitioners found that family physicians seeing 25 patients in a typical day of outpatient care may have 15 clinical questions, but many are either not pursued or answers are not found [17-22]. Barriers to successful information seeking include limited insight on gaps in knowledge or skill, time constraints, access to information resources, searching ability, perceived attributes of the information sources, critical appraisal skills, and evidence that is incomplete, contradictory, or not applicable to individual patients [23-28]. It is not known whether search strategies that increase the sensitivity and specificity of retrieving clinically relevant research articles from literature databases, or journals that provide evidence synopses are used by health care providers [29,30]. A systematic review of 19 studies reported that the information sources used most often by general practitioners were textbooks such as physician desk references due to ease of access, and asking colleagues who can provide tacit, experiential knowledge to overcome organizational demands and constraints [31,32].

General surgeons care for a considerable proportion of patients with cancer, providing diagnosis, surgical treatment, and follow-up monitoring, and function as a critical link between patients and other cancer experts such as radiation and medical oncologists, and surgical sub-specialists. Thus it is important to investigate and optimize resources and processes associated with evidence seeking and utilization among general surgeons. As generalists who provide services for a wide range of conditions in both community and academic settings, their information seeking and utilization patterns may be similar to those of family physicians, but different from specialist surgeons who practice primarily in academic settings with greater access to human, information and technologic resources. A single identified study on information seeking by surgeons reported that they most often consult colleagues over other sources [31]. Through a series of interviews with community-based general surgeons we learned that they deal with very complex patient management issues which are not easily addressed by research evidence, and have few formal or informal opportunities for collegial interaction either within or outside of their organizations to discuss patient management issues [33]. In particular, lack of human and technical resources, and of organizational mechanisms to support multidisciplinary interaction needed for decision-making impeded information seeking and application, and the impact of quality improvement efforts [34,35].

To confirm and elaborate on these exploratory findings we surveyed general surgeons in the population from which interview participants were selected. Specifically, we collected data on the information resources that general surgeons use to address cancer-related questions, the factors that influence and challenge information seeking and use associated with cancer patient care, and the resources or strategies that they believe would address these challenges.

## Methods

### Approach

A survey strategy based on standard descriptive research methods was used to explore factors influencing information seeking and utilization among general surgeons in Ontario, Canada, and suggested improvements [36]. This involved quantitative and qualitative analysis of responses to closed and open questions in a mailed, cross-sectional questionnaire. Despite the fact that physicians are known to under-report information needs and over-report information utilization [37], this approach was considered appropriate as a preliminary step to explore the relative contribution, and interaction of system infrastructure with individual behaviour, and identify potential solutions that could be evaluated in future research. Ethical approval for this study was granted by Sunnybrook Health Sciences Centre.

### Sampling

Contact information for general surgeons was obtained from the Canadian Medical Directory (n = 728). Eligible surgeons included practicing surgeons with privileges at community and academic acute care hospitals in Ontario, Canada and a primary specialty of general surgery not affiliated with our research group (-52). Surgeons were excluded if they possessed a sub-specialty suggestive of limited cancer management such as cardiac, head and neck, thoracic, trauma, urologic, or vascular surgery or endoscopy practice (-232), or responded that they did not treat cancer patients (-59). A total of 385 general surgeons were considered eligible.

### Data collection

Based on research describing health professional information seeking and utilization [38], a questionnaire was developed to elicit information on information need (type of cancer, task/issue, question importance), information seeking (source, frequency of use), and reasons for use (familiarity, prior success, speed of access, applicability to patients); individual attributes (sex, age, academic or community setting) and organizational or system features (resource availability, important/common challenges to delivering care), and suggested strategies for supporting cancer care delivery (See Additional file 1). Most questions were closed with nominal (two or more categories) or ordinal response options (five-point scale). All closed questions allowed respondents to add, and rate additional relevant items not already listed. Questions about most common problems faced when caring for cancer patients and the resources or strategies to address each were open-ended.

The questionnaire was not tested for psychometric properties because the purpose of this survey was exploratory and descriptive, and not analytic. However, the questionnaire was pilot-tested for face validity with four general surgeons who were subsequently not surveyed. They were asked to complete the questionnaire and provide feedback on format, clarity and meaning of questions, instructions and response options. Their suggestions were all incorporated in the questionnaire, which was then reviewed for the same issues by all co-investigators, who suggested further minor modifications to wording and response options.

Based on research evidence for increasing survey response rates, the questionnaire was mailed with an addressed, stamped return envelope, and a personalized cover letter identifying academic affiliation of the researchers, and endorsement by both the Ontario agency overseeing cancer services, and the Ontario professional association for general surgeons; a second package was mailed after two weeks to non-responders; and a third package was mailed to non-responders after another two-week period [39,40]. The names of those returning a completed survey were entered into a draw for a $1000 gift certificate. Initial distribution took place on March 26, 2007 and the third reminder package was distributed on May 1, 2007.

### Data analysis

Survey responses were entered by one individual into an Access database with validation rules to minimize data entry errors. Double data entry was performed by a second individual by entering a random sample of 10% of the surveys into a replicated Access database. The two databases were compared and no consistent errors were noted. Statistically significant differences in characteristics (sex, age, setting) between responders and non-responders were calculated with the chi square test. Questionnaire responses were analyzed for the entire group using summary statistics (frequency, proportion). Statistical significance of differences among sub-groups (age, setting) was established with the Kruskal Wallis test or Mann Whitney U test for information need and resources used, and with the chi square test for information seeking and reasons that particular resources were used. Chi square was reported with a continuity correction for categories where counts were fewer than five. Age was categorical for the Kruskal Wallis test (30–39, 40–49, 50–59, 60+) and nominal for the chi square test (<50, ≥ 50) to optimize cell counts. All statistical analyses were performed in SPSS 16.0.

Open-ended responses were examined thematically using standard qualitative analysis methods and a grounded approach, meaning ideas were inductively extracted from the responses [41,42]. This involved repeated reading to identify key themes, developing of codes to reflect themes, applying thematic codes to all relevant responses, and grouping of responses by theme. Several strategies were employed for sampling (population-based, identification of limitations), analysis (data examined independently by three individuals) and interpretation (reporting of findings with anonymous identifier codes to illustrate both congruent and divergent themes, comparison of findings with other research) to enhance the reliability and validity of these findings.

## Results

### Respondents

The overall response rate was 44.2% (170/385). Responses were received from all 14 health regions ranging from 16.1% (5/31) to 77.8% (7/9). Responders included a higher proportion of surgeons in academic settings compared with non-responders (p = 0.024). There were no significant differences between responders and non-responders by sex or year from graduation (Table 1). Respondents are involved in breast and colorectal cancer surgery primarily. They reported the following proportion of practice devoted to breast cancer: less than 10% (50.0%), 10–25% (27.6%), 26–50% (14.1%), and more than 51% (8.2%) and colorectal cancer: less than 10% (31.8%), 10–25% (41.8%), 26–50% (14.7%), and more than 51% (11.8%). While there was no significant difference in the range of proportion of practice devoted to colorectal cancer surgery by setting (p = 0.912), significantly more surgeons in community settings manage lower volumes of breast cancer (up to 25% of practice) (p < 0.001). The majority of surgeons reported that less than 10% of their practice was devoted to gastric (92.9%), melanoma (92.4%) or hepatopancreatobiliary cancer (89.4%).

**Table 1:** Comparison of responders to non-responders

Subgroup	Eligible	Responders	%	Nonresponders	%	p-value
Sex						
male	332	141	42.47	191	57.53	0.129
female	53	29	54.72	24	45.28	
Graduation						
≤ 1979	134	54	40.30	80	59.70	0.114
1980–1989	114	60	52.63	54	47.37	
≥ 1990	137	56	40.88	81	59.12	
Setting						
academic	112	59	52.68	53	47.32	0.024
community	273	111	40.66	162	59.34	
Health region						
1	19	7	36.84	12	63.16	0.022
2	37	18	48.65	19	51.35	
3	14	10	71.43	4	28.57	
4	50	21	42.00	29	58.00	
5	9	7	77.78	2	22.22	
6	29	11	37.93	18	62.07	
7	61	33	54.10	28	45.90	
8	26	12	46.15	14	53.85	
9	39	21	53.85	18	46.15	
10	17	8	47.06	9	52.94	
11	31	5	16.13	26	83.87	
12	15	5	33.33	10	66.67	
13	27	9	33.33	18	66.67	
14	11	3	27.27	8	72.73	

Total	385	170		215		

### Information needs

The three top-ranked tasks or issues giving rise to at least some uncertainty (Table 2) when managing cancer patients were human and technologic resource constraints (60.4%), comorbid conditions (56.4%) and other patient factors such as age and physiology (51.8%). In contrast, the top-ranked clinical tasks giving rise to uncertainty that would reportedly trigger information seeking were chemotherapy or radiotherapy treatment (92.4%), pathology (92.9%), and surgical approach or technique (87.4%), followed closely by lack of or conflicting evidence (86.5%) and tumour stage (84.4%). These views did not differ significantly by age or setting of care.

**Table 2:** Factors contributing to information needs and information seeking

Factor	Degree of uncertainty		Uncertainty triggers information seeking	
	N	%	N	%

Resource availability (human/technologic)	99	60.4	111	73.0
Comorbid conditions	93	56.4	122	77.7
Patient factors such as age or physiology	86	51.8	107	68.6
Evidence, unaware, lacking or conflicting	74	45.7	134	86.5
Tumour stage	64	38.6	130	84.4
Patient safety	60	36.8	113	73.9
Chemotherapy or radiotherapy	59	35.8	146	92.4
Patient preferences	56	33.7	88	58.3
Ethical issues	42	25.8	105	68.6
Pathology	40	23.8	145	92.9
Legal issues	35	21.2	104	68.9
Surgery	21	12.7	132	87.4

### Information seeking

The three sources of information used by surgeons most frequently when faced with clinical uncertainty (Table 3) were local surgical colleagues (84.6%), other local colleagues (82.2%), and the Internet (81.1%), followed closely by office journals (77.6%). This did not differ significantly by age or setting of care. Surgeons in community settings were more likely to phone (p = 0.007), or refer to external specialists (p = 0.018). A larger proportion of community surgeons reported never taking part in intra- or inter-departmental meetings, or cancer conferences. Those that did were more likely to attend on a monthly basis compared with surgeons in academic settings who reported higher rates of weekly participation (p < 0.001).

**Table 3:** Sources used during information seeking

Source	Frequency of use	
	N	%

Local colleague, surgeon	143	84.6
Local colleague, clinician	139	82.2
Internet (journals, guidelines)	137	81.1
Journal in office	128	77.6
Intradepartment meeting	119*	70.4
Textbook in office	100	59.9
Interdepartment meeting	99*	59.3
Cancer conferences (local, regional)	86*	50.9
Refer to external specialist	70*	41.9
Phone external specialist	68*	40.5
Hospital library	63	37.7
Computer decision aid	37	22.7
Librarian	19	11.6

* p < 0.05 by Mann Whitney U test two-tail significance for setting of care

When asked to select the reasons for using various sources of information (Table 4), local surgical (64.7%) and other colleagues (57.6%) and office textbooks (59.4%) and journals (48.2%) were ranked most familiar; local surgical (50.6%) and other colleagues (43.5%) and either phone calls (41.8%) or referrals to external specialists (42.4%) were highest ranked for prior success; the Internet, defined as any resource providing access to evidence such as journals or guidelines (57.1%), local surgical colleagues (51.2%), and office textbooks (58.2%) or journals (48.2%) offered speediest access to cancer-related information; and cancer conferences (35.9%) and local surgical colleagues (34.1%) provided information considered to be the most applicable to patient care. Older surgeons more often used the hospital library (p = 0.042) or interdepartmental meetings (p = 0.011) due to familiarity, and fewer considered office journals to be applicable (p = 0.046) compared with younger surgeons. Community-based surgeons more often chose to refer to external specialists (p = 0.003) or consult computer decision aids (p = 0.009) based on prior success. Academic surgeons most often used interdepartmental meetings for this reason (p = 0.017). Speedy access prompted more academic surgeons to consult office journals (p = 0.009) while community surgeons more often reported applicability as the reason for using office textbooks (p = 0.044).

**Table 4:** Reasons for use of information resources

Source	Reasons for use							
	Familiar		Prior success		Quick access		Most applicable	
	N	%	N	%	N	%	N	%

Textbook in office	101	59.4	64	37.6	99*	58.2	35	20.6
Journal in office	82	48.2	63	37.1	82**	48.2	37	21.8
Hospital library	50*	29.4	39	22.9	45	26.5	21	12.4
Librarian	25	14.7	24	14.1	20	11.8	13	7.6
Local colleague, surgeon	110	64.7	86	50.6	87	51.2	58	34.1
Local colleague, clinician	98	57.6	74	43.5	71	41.8	47	27.6
Phone external specialist	58	34.1	71	41.8	25	14.7	43	25.3
Refer to external specialist	65	38.2	72**	42.4	17	10.0	46	27.1
Computer decision aid	16	9.4	17**	10.0	22	12.9	14	8.2
Internet (journals, guidelines)	70	41.2	68	40.0	97	57.1	42	24.7
Intradepartment meeting	72	42.4	45	26.5	36*	21.2	34	20.0
Interdepartment meeting	56*	32.9	47**	27.6	33	19.4	30	17.6
Conferences (local, regional)	70	41.2	67	39.4	29	17.1	61	35.9

* p < 0.05 by Chi square test two-tail significance for age.

** p < 0.05 by Chi square test two-tail significance for setting of care

**Figure 1:**
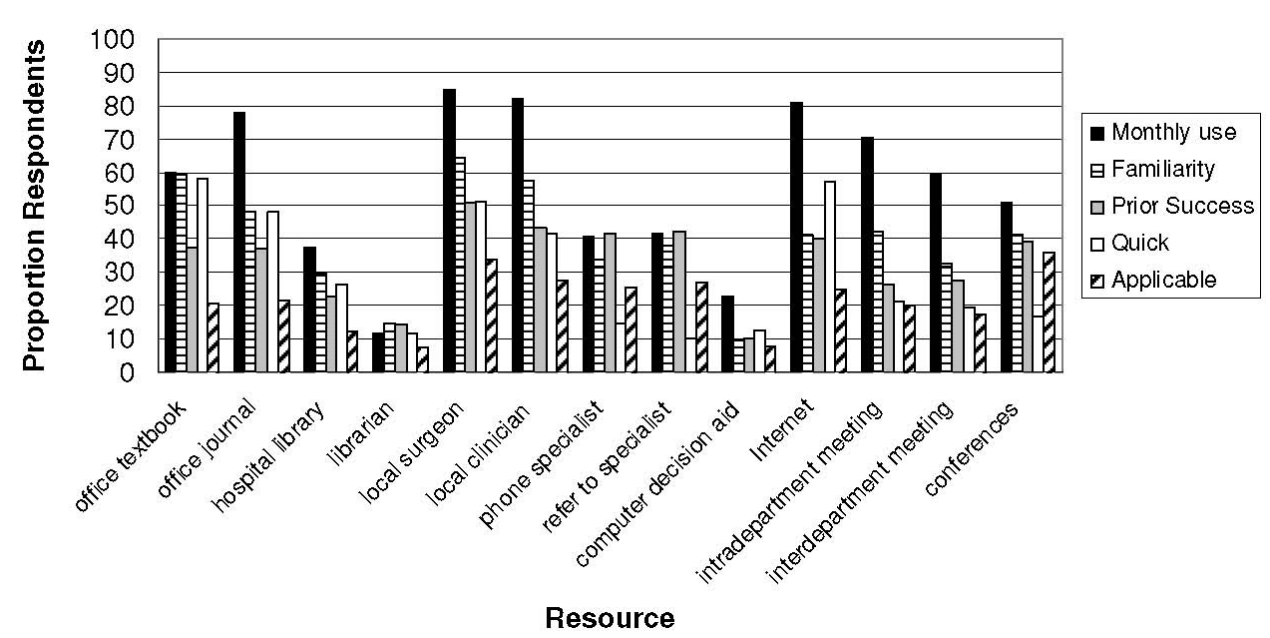
**Reported use and reasons for use of information resources**.

Sources of information used at least monthly were considered according to reasons for use (Figure 1). The single resource used most frequently by the majority of respondents, local surgical colleagues, was ranked highly for each of familiarity, prior success, speedy access and applicability to patients. Familiarity and prior success contribute to use of the second most frequently used resource, other local colleagues. The third most used resource, the Internet, was viewed as readily accessible. Notably, most resources were not considered by the majority of surgeons to provide information that was applicable to cancer patient care, including local surgical colleagues, thought by 34.1% of respondents to provide relevant information, despite being the most frequently consulted resource.

### Cancer management challenges

In response to an open-ended question, 74% (126/170) of respondents listed up to three important concerns faced when caring for cancer patients, and suggested solutions (Table 5). These were organized into eight thematic categories. The majority of comments were related to barriers to diagnosing and staging cancer, lack of operative resources, and barriers to coordinating multidisciplinary care. Together these accounted for 76.0% (204/268) of all reported issues. Increased funding for resources was the most frequently mentioned solution for these concerns (82/167, 49.1%). The availability of evidence to support cancer care decision making was named by 19 individuals (19/268, 7.1%). Greater production of guidelines was recommended as a possible solution by four individuals. Thus information seeking and utilization was not viewed as an important challenge compared with limitations in system infrastructure and communication with colleagues to coordinate care delivery.

**Table 5:** Cancer care concerns and suggested solutions

Concerns (n, % respondents)	Issues (n respondents)	Suggested solutions (n respondents)
Barriers to diagnosing and staging cancer (82, 30.6)	CT/MRI/ERUS (22) Endoscopy (21) Core biopsy/breast imaging (19) Access to/timely radiology (17) Referral by family physicians (3)	Funding for more resources (33) Establish and monitor benchmarks (6) Prioritize cancer cases (5) Centralized workup facility (4) Improved coordination (3) Patient education (1) Privatize (1) Colorectal cancer screening program (1)
Lack of operative resources (67, 25.0)	OR time (53) Lack of surgical beds (5) Nuclear medicine/equipment for SLNB (4) Anesthesia shortage (2) Lack of nurses (1) Old laparoscopic equipment (1) Surgery assistants (1)	Funding for more resources (24) Prioritize cancer cases (11) Lobby/increase public awareness (2) Weekend operating time (1) Privatize (1) Create non-hospital surgical centres (1) Clinical model (refer to group) (1)
Barriers to coordinating multidisciplinary care (55, 20.0)	Coordination of care (23) Referral to specialists (16) Proximity to cancer specialists (6) Challenge of participating in tumor boards (6) Patient retention at large centres (2) Challenges of organizing clinical trials (2)	Regional integration/coordination services (7) Funding for more specialists (5) Oncology electronic medical record (5) Communication to referring offices (5) Radiation oncologist visits satellite clinic (3) Open new radiation facilities (3) Organized implementation tumor boards (3) List of available specialists (2) Patient-centred care pathway (1)
Lack of data to guide care delivery (20, 7.5)	Lack of/applicability of evidence (19) Timely performance measurement (1)	Patient-specific guidelines (4) Funding for more resources (3) Tumor boards (2) Develop cancer-specific programs (2)
Access to pathology (15, 5.6)	Delayed pathology (15)	Funding for more resources (8) Establish and monitor benchmarks (1)
Need for patient support resources (12, 4.5)	Navigation (5) Information (5) Shared decision making tools (2)	Funding for the development of information resources (5)
Heavy workload (10, 3.7)	Overwhelmed (9) Patients with no family physician (1)	Nurse practitioners/GP oncologists (5) Funding for more surgeons (4) Prioritize cancer patient access to family physicians (1)
Need for opportunities to develop skills (7, 2.6)	Training for practicing surgeons (7)	Increase mentorship opportunities (3)

CT computed tomography scan; MRI magnetic resonance imaging; ERUS endorectal ultrasound; SLNB sentinel lymph node biopsy

## Discussion

The overall objective of this research was to understand how general surgeons could be better supported to undertake decision making for patients with cancer. In previous exploratory work we learned that general surgeons care for patients with complex cancer problems [33]. This population-based survey confirms that general surgeons experience clinical uncertainty at an informational level due to the complexity of care required for cancer patients who have comorbid conditions and other physiologic factors that confound management. Since these uncertainties may not be directly addressed by searching for available research evidence, respondents most often turn to local surgical colleagues, as was found to be the case among family physicians [31,32]. Perhaps because research on information seeking and utilization by family physicians was conducted in the 1980s and 1990s when the Internet was not widely available, in contrast to family physicians, general surgeons appear to frequently use the Internet [17-22,37]. These findings were similar for surgeons of younger and older age, and practicing in both community and academic settings. Surgeons also experience clinical dilemmas at a logistical level related to human and technologic constraints in the health care sector that challenge the appropriate and coordinated care of complex cancer cases. Community-based surgeons reported no, or limited interaction with colleagues for the discussion of patient care issues, and frequently refer patients to other centres for diagnostic, surgical and oncologic services. This was also identified in our previous study [33].

While surgeons consulted the Internet and colleagues, neither was considered highly applicable to the expressed informational and logistical decision-making needs associated with complex management issues. Among a population-based sample of 15,626 American patients with cancer, 68.7% had comorbidity, and 32.6% had two or more comorbid conditions [43]. These rates were higher in the elderly, smokers and those with lower socioeconomic status. Hence, the complexity of decision-making for a considerable number of cancer patients might not be entirely solved by either developing more guidelines, or providing surgeons with informatics training or tools since available evidence may not be applicable to individual patients with complex indications. Instead, qualitative information collected by this survey suggests that better access to, and coordination of multidisciplinary decision-making could address many of the identified informational and logistical problems.

Recommendations of an informational nature included development of a cancer care medical record to provide electronic access to consolidated patient information. Electronic health records may not be prevalent in many settings, nor shared across the different settings that are involved in delivering care to individual patients [44]. Surveys of Canadian and American hospitals found that few have implemented such systems [45,46]. Research investigating the use of patient held records for patients with cancer found that there was a low level of use, perhaps due to lack of agreement between patients and health professionals regarding their function [47-50]. Many respondents highlighted the need for more efficient communication of tests and treatment results to referring surgeons from cancer clinics. Lack of referral reply, delay in receiving the consultant's reply letter, and insufficient detail in reply letters are common concerns expressed by surgeons elsewhere [51]. Content analysis of both referral and reply letters has shown that their quality and comprehensiveness could be improved [51-53]. Communication could be enhanced with the use of structured letter templates that facilitate more consistent inclusion of key patient and educational information to referring doctors [53,54].

Recommendations to address logistical issues such as surgeon interaction with other health care professionals involved in diagnosing, staging or treating cancer patients included development of centralized cancer diagnostic facilities or satellite cancer clinics on a regional basis. We conducted a systematic review of diagnostic assessment units for cancer [55]. While evidence was limited, they appear to reduce time to diagnosis. Another systematic review based on nine studies found that specialist outreach clinics improved access, health outcomes, more efficient and guideline-consistent care, and less use of inpatient services [56]. Several respondents recommended greater use of multidisciplinary cancer conferences (MCCs), or tumour boards [57]. We found that videoconferencing could be used to successfully involve community-based surgeons in MCCs [58], and subsequently evaluated an MCC that regularly engages surgeons affiliated with six community hospitals in one health region, and oncologists from the closest cancer centre [33]. Physicians thought that collegial interaction improved awareness of current evidence, patient satisfaction with treatment plans, appropriate care delivery, and continuity of care. Given that most of our survey respondents were frequent Internet users, further development of this platform to support multidisciplinary consultation and care delivery is warranted since it was used successfully to support MCCs in Germany [59].

The results of this survey are limited by self-report bias inherent in survey methods, and the 44.2% response rate which is lower than the mean response rate of 54% for published surveys of physicians [40,41]. Non-response to surveys is a problem if the respondents differ in a meaningful way from non-respondents. We found no difference between responders and non-responders by sex or graduation date (proxy for age). Although some regions were less well represented than others, for reasons we cannot identify based on this data, we did achieve responses from all 14 health regions, thusly accounting for geographic factors that might impact information seeking. While general surgeons in academic settings were over-represented among respondents, we received information from 59 academic and 111 community surgeons, providing sufficient power to find no statistically significant differences in our key results according to this factor. Hence, we believe that our findings are generalizable to the general surgeon population from which our sample was drawn. They may be less relevant to general surgeons in other jurisdictions where resource constraints and the organization of cancer care services may be different from those in Ontario. Furthermore, we examined perceived informational needs, and not specific patient care decisions made, and their correspondence with evidence of appropriate care. Since physicians are known to under-estimate their need for knowledge [23,37], it may be that current information seeking behaviour among this population inadequately makes use of existing information resources, but this would need to be addressed in further studies. Ongoing research should evaluate the implementation and benefit of various multidisciplinary care models, including concurrent (outreach clinics, centralized diagnostic centres, MCCs, telemedicine, Internet networking) and consecutive (improved referral communication, electronic health records, patient-held medical records) mechanisms to assess their cost-effectiveness.

## Conclusion

This study found that, regardless of age or setting of care, general surgeons consult a variety of informational resources, most frequently colleagues, although none are considered to adequately solve clinical uncertainty associated with the complexity of many cancer patients. Decision-making is challenged by both informational and logistical issues related to the coordination of multidisciplinary care. This suggests that limitations in the organization of multidisciplinary care leading to suboptimal exchange of information among involved health care professionals may, in part, contribute to variable compliance with what is considered to be appropriate care according to current guidelines. There are many different mechanisms by which multidisciplinary care can be achieved but none have been comprehensively evaluated. Ongoing research will develop and evaluate mechanisms by which to support peer interaction for routine decision making and continuing professional development.

## Competing interests

The authors declare that they have no competing interests.

## Authors' contributions

ARG conceptualized and designed the study, obtained funding, coordinated all aspects, collected and analyzed data, interpreted findings, and prepared the manuscript. FCW, DD, RSM and DRU assisted with design of the study, interpretation of data, and review of the manuscript. All authors read and approved the final manuscript.

## Pre-publication history

The pre-publication history for this paper can be accessed here:



## Supplementary Material

Additional file 1 **Survey instrument.** Questionnaire used to collect data from general surgeons on health professional information seeking and utilization patterns. Click here for file
