# Supplementary material for: Challenges in multidisciplinary cancer care among general surgeons in Canada
Source: BMC Med Inform Decis Mak. 2008 Dec 22;8:59. doi: 10.1186/1472-6947-8-59 (PMC2631026; doi:10.1186/1472-6947-8-59)
Supplement: Additional file 1 — Survey instrument. Questionnaire used to collect data from general surgeons on health professional information seeking and utilization patterns. [file 1472-6947-8-59-S1.doc]

**A. BACKGROUND INFORMATION**

1) About you

| Sex | 🞎_1_ male | 🞎_2_ female |  |  |  |
| --- | --- | --- | --- | --- | --- |
| Age | 🞎_1_ 20-29 | 🞎_2_ 30-39 | 🞎_3_ 40-49 | 🞎_4_ 50-59 | 🞎_5_ 60+ |
| Setting | 🞎_1_ community | 🞎_2_ teaching / academic |  |  |  |

| **2) In the future, would you prefer to complete a similar questionnaire on a web site rather than in print form?** | 🞎_2_ yes | 🞎_1_ no |
| --- | --- | --- |

**3) What type of cancer patients do you treat?** *Check all that apply*

| Type of Cancer | Estimated proportion of **total** surgical practice (%) | | | | |
| --- | --- | --- | --- | --- | --- |
|  | <10% | 10-25% | 26-50% | 51-75% | 75%+ |
| Breast | 🞎_1_ | 🞎_2_ | 🞎_3_ | 🞎_4_ | 🞎_5_ |
| Colorectal | 🞎_1_ | 🞎_2_ | 🞎_3_ | 🞎_4_ | 🞎_5_ |
| Gastric | 🞎_1_ | 🞎_2_ | 🞎_3_ | 🞎_4_ | 🞎_5_ |
| Genitourinary | 🞎_1_ | 🞎_2_ | 🞎_3_ | 🞎_4_ | 🞎_5_ |
| Gynecologic | 🞎_1_ | 🞎_2_ | 🞎_3_ | 🞎_4_ | 🞎_5_ |
| Head and Neck | 🞎_1_ | 🞎_2_ | 🞎_3_ | 🞎_4_ | 🞎_5_ |
| Hepatobiliary-Pancreatic | 🞎_1_ | 🞎_2_ | 🞎_3_ | 🞎_4_ | 🞎_5_ |
| Lung | 🞎_1_ | 🞎_2_ | 🞎_3_ | 🞎_4_ | 🞎_5_ |
| Melanoma | 🞎_1_ | 🞎_2_ | 🞎_3_ | 🞎_4_ | 🞎_5_ |

**B. UNCERTAINTIES IN CANCER PATIENT MANAGEMENT**

| Issue | **1) To what degree do these issues contribute to uncertainty in your management of cancer patients?** C*ircle the best response, where 1= very little and 5=very much; NS=not sure* | | | | | | **2) Would uncertainty prompt you to seek information to assist in decision-making for this issue?** |
| --- | --- | --- | --- | --- | --- | --- | --- |
|  | Very little | |  | Very much | | NS |  |
| Surgical approach or technique | 1 | 2 | 3 | 4 | 5 | NS | 🞎_2_ yes 🞎_1_ no |
| Chemotherapy / Radiation Therapy | 1 | 2 | 3 | 4 | 5 | NS | 🞎_2_ yes 🞎_1_ no |
| Interpretation of pathology | 1 | 2 | 3 | 4 | 5 | NS | 🞎_2_ yes 🞎_1_ no |
| Lack of / conflicting evidence | 1 | 2 | 3 | 4 | 5 | NS | 🞎_2_ yes 🞎_1_ no |
| Expressed patient preferences | 1 | 2 | 3 | 4 | 5 | NS | 🞎_2_ yes 🞎_1_ no |
| Comorbid conditions | 1 | 2 | 3 | 4 | 5 | NS | 🞎_2_ yes 🞎_1_ no |
| Patient factors (i.e. age, physiology) | 1 | 2 | 3 | 4 | 5 | NS | 🞎_2_ yes 🞎_1_ no |
| Tumour factors (i.e. stage, pathology) | 1 | 2 | 3 | 4 | 5 | NS | 🞎_2_ yes 🞎_1_ no |
| Patient safety concerns | 1 | 2 | 3 | 4 | 5 | NS | 🞎_2_ yes 🞎_1_ no |
| Ethical issues | 1 | 2 | 3 | 4 | 5 | NS | 🞎_2_ yes 🞎_1_ no |
| Legal issues | 1 | 2 | 3 | 4 | 5 | NS | 🞎_2_ yes 🞎_1_ no |
| Resource availability (i.e. human, technologic) | 1 | 2 | 3 | 4 | 5 | NS | 🞎_2_ yes 🞎_1_ no |
| Other: *please name* |  |  |  |  |  |  |  |
|  | 1 | 2 | 3 | 4 | 5 | NS | 🞎_2_ yes 🞎_1_ no |
|  | 1 | 2 | 3 | 4 | 5 | NS | 🞎_2_ yes 🞎_1_ no |

| Source | **1) How frequently do you consult these sources for information on cancer patient management?** | | | | | **2) Which reasons influence your use of these information sources?** *Check all that apply* | | | |
| --- | --- | --- | --- | --- | --- | --- | --- | --- | --- |
|  | daily | weekly | monthly | yearly | never | familiar with source | prior success with source | source quickly accessible | information most applicable to patient care |
| Textbook in office/clinic | 🞎_1_ | 🞎_2_ | 🞎_3_ | 🞎_4_ | 🞎_5_ | 🞎_2_ | 🞎_2_ | 🞎_2_ | 🞎_2_ |
| Journals in office/clinic | 🞎_1_ | 🞎_2_ | 🞎_3_ | 🞎_4_ | 🞎_5_ | 🞎_2_ | 🞎_2_ | 🞎_2_ | 🞎_2_ |
| Textbooks/journals in hospital library | 🞎_1_ | 🞎_2_ | 🞎_3_ | 🞎_4_ | 🞎_5_ | 🞎_2_ | 🞎_2_ | 🞎_2_ | 🞎_2_ |
| Librarian | 🞎_1_ | 🞎_2_ | 🞎_3_ | 🞎_4_ | 🞎_5_ | 🞎_2_ | 🞎_2_ | 🞎_2_ | 🞎_2_ |
| Local colleague, surgeon | 🞎_1_ | 🞎_2_ | 🞎_3_ | 🞎_4_ | 🞎_5_ | 🞎_2_ | 🞎_2_ | 🞎_2_ | 🞎_2_ |
| Local colleague, other clinician | 🞎_1_ | 🞎_2_ | 🞎_3_ | 🞎_4_ | 🞎_5_ | 🞎_2_ | 🞎_2_ | 🞎_2_ | 🞎_2_ |
| Phone call to external specialist | 🞎_1_ | 🞎_2_ | 🞎_3_ | 🞎_4_ | 🞎_5_ | 🞎_2_ | 🞎_2_ | 🞎_2_ | 🞎_2_ |
| Refer patient to external specialist | 🞎_1_ | 🞎_2_ | 🞎_3_ | 🞎_4_ | 🞎_5_ | 🞎_2_ | 🞎_2_ | 🞎_2_ | 🞎_2_ |
| Computer program (i.e. decision aids) | 🞎_1_ | 🞎_2_ | 🞎_3_ | 🞎_4_ | 🞎_5_ | 🞎_2_ | 🞎_2_ | 🞎_2_ | 🞎_2_ |
| Internet (i.e. journals, guidelines) | 🞎_1_ | 🞎_2_ | 🞎_3_ | 🞎_4_ | 🞎_5_ | 🞎_2_ | 🞎_2_ | 🞎_2_ | 🞎_2_ |
| Intra-departmental meetings (surgery) | 🞎_1_ | 🞎_2_ | 🞎_3_ | 🞎_4_ | 🞎_5_ | 🞎_2_ | 🞎_2_ | 🞎_2_ | 🞎_2_ |
| Inter-departmental meetings (other clinicians) | 🞎_1_ | 🞎_2_ | 🞎_3_ | 🞎_4_ | 🞎_5_ | 🞎_2_ | 🞎_2_ | 🞎_2_ | 🞎_2_ |
| Cancer conferences (local, regional) | 🞎_1_ | 🞎_2_ | 🞎_3_ | 🞎_4_ | 🞎_5_ | 🞎_2_ | 🞎_2_ | 🞎_2_ | 🞎_2_ |
| Other: *please name* |  |  |  |  |  |  |  |  |  |
|  | 🞎_1_ | 🞎_2_ | 🞎_3_ | 🞎_4_ | 🞎_5_ | 🞎_2_ | 🞎_2_ | 🞎_2_ | 🞎_2_ |
|  | 🞎_1_ | 🞎_2_ | 🞎_3_ | 🞎_4_ | 🞎_5_ | 🞎_2_ | 🞎_2_ | 🞎_2_ | 🞎_2_ |

**Please name the three most important/common problems you face in caring for cancer patients, and the resources or strategies that you believe would best address each issue.**

| Problem | Strategy |
| --- | --- |
| 1. |  |
| 2. |  |
| 3. |  |

**Do you have any further general comments about factors that would help you to care for cancer patients?**

**THANK YOU**
